# Seasonal movements of Bronze Age transhumant pastoralists in western Xinjiang

**DOI:** 10.1371/journal.pone.0240739

**Published:** 2020-11-04

**Authors:** Peter Jia, Gino Caspari, Alison Betts, Bahaa Mohamadi, Timo Balz, Dexin Cong, Hui Shen, Qi Meng

**Affiliations:** 1 Department of Archaeology, University of Sydney, Sydney, NSW, Australia; 2 School of Culture and History, Henan University, Kaifeng, China; 3 Institute of Archaeological Sciences, University of Bern, Bern, Switzerland; 4 State Key Laboratory of Information Engineering in Surveying, Mapping and Remote Sensing, Wuhan University, Wuhan, China; 5 Institute of Archaeology, Chinese Academy of Social Science, Beijing, China; 6 University of Chinese Academy of Sciences, Beijing, China; Washington University in Saint Louis, UNITED STATES

## Abstract

The paper explores seasonal movements of Bronze Age mobile pastoralists in the western Tianshan mountainous region of Xinjiang, China. Fieldwork by a team from the Institute of Archaeology of the Chinese Academy of Social Science (CASS) and the University of Sydney, Australia have identified cyclical land use practices associated with the Andronovo cultural complex. Their pattern of seasonal movements has been reconstructed through ethnographic studies and analysis of modern snow and grass cover. Using this detailed combination of data, the study defines requirements for seasonal pastures–winter, summer and spring/autumn–and shows a clear correlation between modern land use and seasonal patterns of movement in the Bronze Age.

## 1. Introduction

Western Xinjiang is a land of mountains where the narrow ridge of the Tianshan is split in two by the Yili River. The high steep-sided peaks are permanently glaciated, forming numerous rivers cutting deep valleys down to the desert below. The upper slopes below the snowline are forested, with open patches of grassland. With the exception of the Yili Valley, rainfall is low, and the land is not well suited to agriculture, although dry farming is possible in some particularly favourable places. Irrigation agriculture is practiced today at the foot of the mountains, exploiting permanently flowing rivers fed by snow and glacial meltwater. Historically, the main economic strategy has been transhumant pastoralism, taking advantage of the extreme ranges of altitude and the rich seasonal grassland pastures. This is still the case today where Kazakhs and Mongols, who have historically migrated into this region, herd sheep, goat, cattle and horses in a round of seasonal movement. Archaeologically, this lifestyle can be traced back in various forms to the Bronze Age when the first herders moved into the western Tianshan from the Eurasian steppe.

Transhumant pastoralism is characterized by regular movement of herders and their livestock between fixed points to exploit the seasonal availability of pastures [1]. In western Xinjiang, annual movement is vertical and typically fairly short range, generally from 20–200 km. Although the herds provide the main basis of the economy for most pastoralists in the region, almost all of them engage to a greater or lesser extent in limited agriculture, depending on the environment in which they operate. Contemporary pastoralists in the lush Yili Valley cultivate quite large numbers of fields, both for fodder and for cash or food crops, but in the more arid Bortala Valley, agriculture is relatively limited. Going back into prehistory, the Eurasian Bronze Age is widely characterised as being based on a pastoral economy, but little work has been carried out on the precise nature of that economy beyond identification of the animals and plants that supported it. In particular, few studies have been undertaken on the nature of seasonal movement in relation to economic management, a notable exception being the work of Frachetti [2] in the Semirech’ye region of eastern Kazakhstan. The aim of this paper is to reconstruct models of seasonal movement for Bronze Age pastoralists in western Xinjiang. The archaeological evidence, while extensive, is insufficient to determine the structure of seasonal movements with any degree of precision, nor is the reasoning behind choices for camp sites clearly identifiable. To model these for the archaeological record, this study uses data drawn from ethnographic studies and analysis of modern snow and grass cover. Fieldwork permission (No. 350) was issued by the Chinese State Bureau of Relics to D. Cong (3196027).

## 2. The Bronze Age in Xinjiang

The first Bronze Age peoples appeared in Xinjiang in the 3^rd^ millennium BCE, mobile pastoralists superseding, and probably blending with, the last communities practicing age-old lifestyles of hunting, fishing and foraging who had dominated the wider region since the Palaeolithic. Bronze Age groups first appeared in the northern part of the Inner Asian Mountain Corridor bordering western Xinjiang when the eastward spread of Yamnaya peoples from beyond the Ural Mountains created the rise of a local Early Bronze Age culture, the Afanasievo, in the Altai Mountains. Afanasievo people in the Altai-Sayan region are genetically indistinguishable from Yamnaya populations, confirming an eastward expansion across the steppe [3]. Afanasievo groups are also linked to the oldest known evidence for dairy consumption in the eastern Eurasian steppe, suggesting that human migration into the Altai was associated with the introduction of domestic livestock [4]. Although a few Afanasievo burials have recently been reported in western Xinjiang [5–7], the first strong evidence for Bronze Age groups there appears with the Qiemu’erqieke (Chemurchek, Khemtseg) population in the north of Xinjiang [8] and Xiaohe/Gumugou to the south [9]. The Qiemu’erqieke culture can be dated broadly from around the mid-3rd millennium BCE, with the tradition as a whole lasting probably up until c. 1700 BCE [10]. Xiaohe/Gumugou dates also from the mid-3rd millennium to the mid-2nd millennium BCE [9]. Qiemu’erqieke and Xiaohe/Gumugou emerged out of Altaic/Mongolian and east Eurasian populations [11–13], while a third early group, the Tianshanbeilu culture, appearing in the oasis of Hami at the eastern end of the Tianshan, had its ancestry to the east in early oasis farming populations in Gansu and the Hexi Corridor [10, 14]. By the early 2nd millennium BCE, new Eurasian agro-pastoralists moved in from the west, occupying some of the same lands as the southerly expansion of Qiemu’erqieke peoples. They spread into the western hills and mountains of Xinjiang, along the Tianshan towards the east, and south into the Pamirs, close to the western end of the Tibetan Plateau. This group shared broad affinity with the loosely defined Andronovo complex that appeared widely across Eurasia in the later Bronze Age [15–17], more specifically the eastern Federovo variant [18].

**Fig 1:**
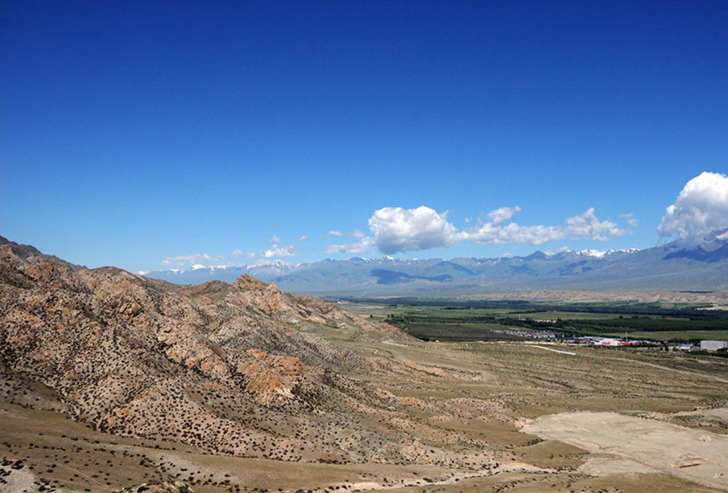
A general view of the Bortala valley looking to the northwest from the town of Wenquan (A. Betts).

A long-term project to conduct a detailed study of Andronovo groups in Xinjiang is in progress under the leadership of a team from the Institute of Archaeology of the Chinese Academy of Social Science (CASS) and the University of Sydney, Australia. The team has been working in Wenquan County, in the Bortala River area, in the far west of Xinjiang [18, 19], particularly at the occupation site and associated burial grounds of Adunqiaolu. The Upper Bortala River, also known as the Wenquan (Hot-Springs) River, originates between the Alatao and Biezhentao Mountains in the western Tianshan, flowing eastward through Wenquan (Hot Springs) County (Fig 1). It then joins the Daheyanzi River and runs out into Aibi (Ebinur) Lake. In the upper reaches of the Bortala River, the river bed lies close to the foothills of the Biezhentao Mountains on the south bank and on the north bank is separated from the Alatao Mountains by a wide band of gently sloping alluvial fans at an elevation of about 2000 m a.s.l. Behind the alluvial fans are low hills, backed in turn by the mountains. The alluvial fans form important areas of open pasture in the semi-arid steppe, drained by a dense network of seasonal streams. The east-west orientation of the valley provides the north bank with maximum exposure to seasonal sunlight. Altitude ranges from c. 1000 in the riverbed to c. 4000 m a.s.l. in the mountains.

Located far from any oceans, the region has a temperate, semi-arid, continental climate. The annual average temperature is around 3.6°C. Temperature extremes range from a maximum of 37.2°C to a minimum of -35.9°C [20]. Generally, the lowest temperature of the year occurs in January, with an average of -15°C, while in July the mean temperature is around 23°C [21]. Rainfall is low with an annual average precipitation rate of c. 200mm, mainly concentrated from May to July, when the region experiences about 60% of the annual total. The other months are much dryer, with monthly precipitation between November and March at usually less than 10mm. The vegetation types in Wenquan county are determined largely by the region’s distinct zones of elevation [22]. In the mountain foothills steppe vegetation is the most common, composed mainly of stipa grasses, including *S*. *caucasica*, *S*. *grandis*, *S*. *orientalis* and *S*. *sareptana*. Plants including sagebrush (*Artemisia frigida*), fetusca grasses (*F*. *ovina*, *F*. *rupicola*), and *Seriphidium gracilescens* are also commonly included in the steppe community. Areas adjacent to and above the steppes are covered with meadow vegetation, with the dominant plants represented by artemesia (*Artemisia subulate*), sedge (*Carex stenocarpa*) and annual meadowgrass (*Poa annua*). Alpine vegetation composed of cushion-like rock jasmine (*Androsace*) dominates on the higher slopes. Forests of Asian spruce (*Picea schrenkiana*) alternate with steppes and meadows, principally on the northern sides of the mountains. Around the Adunqiaolu site, stipa steppe is found widely and constitutes the main landscape. Spruce forest is rare, while in the river valley bottoms poplar (*Populus*) and willow (*Salix*) are common.

As is generally the case with prehistoric pastoralist populations, Bronze Age remains include many cemeteries, but in the Bortala Valley there is also clear evidence of settlement through the presence of stone house footings of distinctive plan. Bronze Age stone-based habitation sites might be far more widespread than previously assumed. This type of monument extends into northern Xinjiang [23, 24], Kazakhstan [25], the Russian Altai and Tuva Republic, although there is an ongoing debate whether smaller versions are connected to seasonal habitation [26] or should rather be interpreted within a ritualistic framework [27, 28]. Archaeologically, investment in construction of stone houses is often taken to indicate permanent settlement, but this is clearly not the case in the Bortala Valley. A body of evidence shows that the houses were used on a rotating seasonal basis; investment in the construction of permanent buildings was worthwhile because the houses were used regularly over many years. This can be proven in various ways, one of the most valuable of which is the recent ethnographic record. Despite the fact that the modern pastoral populations in the region have tents, they construct stone or timber houses which they occupy seasonally, making use of their tents only in the warmer months when they move more widely in search of good pasture. As is the case today, the Andronovo people appear to have constructed houses in differing locations according to the cycles of the seasonal round: for winter, for summer and for the various mid-seasons at either end of the year. Bronze Age houses have been found at high altitudes around 3000 m a.s.l. near the summer campsites of local herders in locations that would be uninhabitable in winter due to deep snow cover and sub-zero temperatures (2016 test pit excavation, unpublished database). Large residential structures have been identified on mountain slopes at the relatively high altitude of 2300 m a.s.l. next to the winter campsites of the modern herding community at Adunqiaolu [18, 29]. As part of the work of the CASS and University of Sydney team in the Bortala Valley, several field seasons have been devoted to study of recent camp sites and herding practices in order to gain a deeper understanding of Bronze Age land use patterns and economic decision making.

## 3. Seasonal movement of modern pastoralists

### 3.1. Seasonal movement

**Fig 2:**
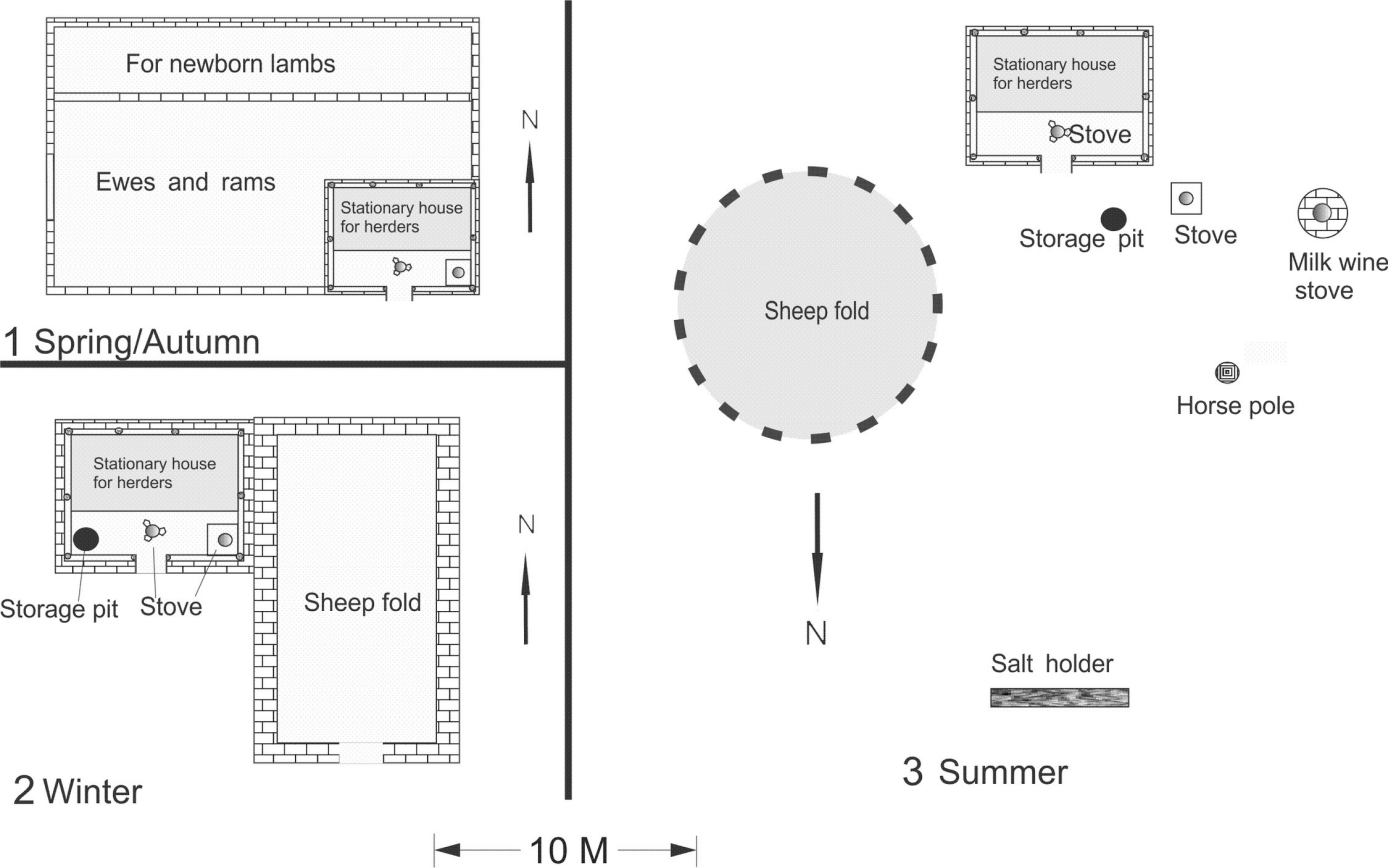
Differences in seasonal camps in the Wenquan area.

Traditional subsistence economies in the valley comprise seasonal transhumant pastoralism, combined with limited cultivation. The modern vertical transhumance pattern is driven by the seasonally oscillating availability of pastureland resources at different altitudes. Transhumance usually includes four major movements correlated with changes through the seasons. There is an annual migration between three seasonal campsites; the local term for the traditional pattern of animal management is “four seasons and three locations”. The herders move their animals following the four seasons, spring, summer, autumn and winter, to provide animals with the best grazing. “Three locations” means that the herders use only three major campsites within three pasturelands over the four seasons, using the same campsite for both spring and autumn. Each campsite is constructed differently, according to the specific needs of the season or seasons in which it is used (Fig 2). The herders move to the spring camps in mid to late March to prepare for lambing. The spring and autumn camps are located in the same place, usually on an open flat low-lying area at around 900–1200 m a.s.l. In these milder seasons the location of adequate grazing takes priority over access to water which may be some distance away. In the low-lying areas the spring grass appears earlier than elsewhere and lasts longer into the autumn. The spring/autumn camp is also near arable land suitable for growing crops or hay, and most herders have a block of land which is used primarily for the cultivation of fodder crops to supplement winter grazing. In early May the move is made to the summer camp when high altitude pastures first start growing. Here the animals are kept until late September. The summer pastures generally lie around the tree line at 3200 m a.s.l. In late September the herds are brought down again to the lowlands to the autumn/spring camp. At this low-lying camp the grazing is available as late as possible into the cold season. In early December the last move of the year is made to the winter camp.

Variations on this type of seasonal pastoral transhumance can be found throughout the Eurasian steppe, in Central Asia, Iran and in the Near East [30–33]. The choice of location and number of seasonal camp sites may differ according to local climate and landscape. Cribb [33, 34] describes a similar pattern of three seasonal camps used over four seasons among western Iranian herders in Luristan. In highland Asia, herders on the Tibetan plateau and some pastoralists along the Himalayan rim may only have two seasonal campsites, for summer and winter respectively [34]. In the Altai Mountains, Kazakh herders may have more than three locations, depending on local conditions [35].

**Fig 3:**
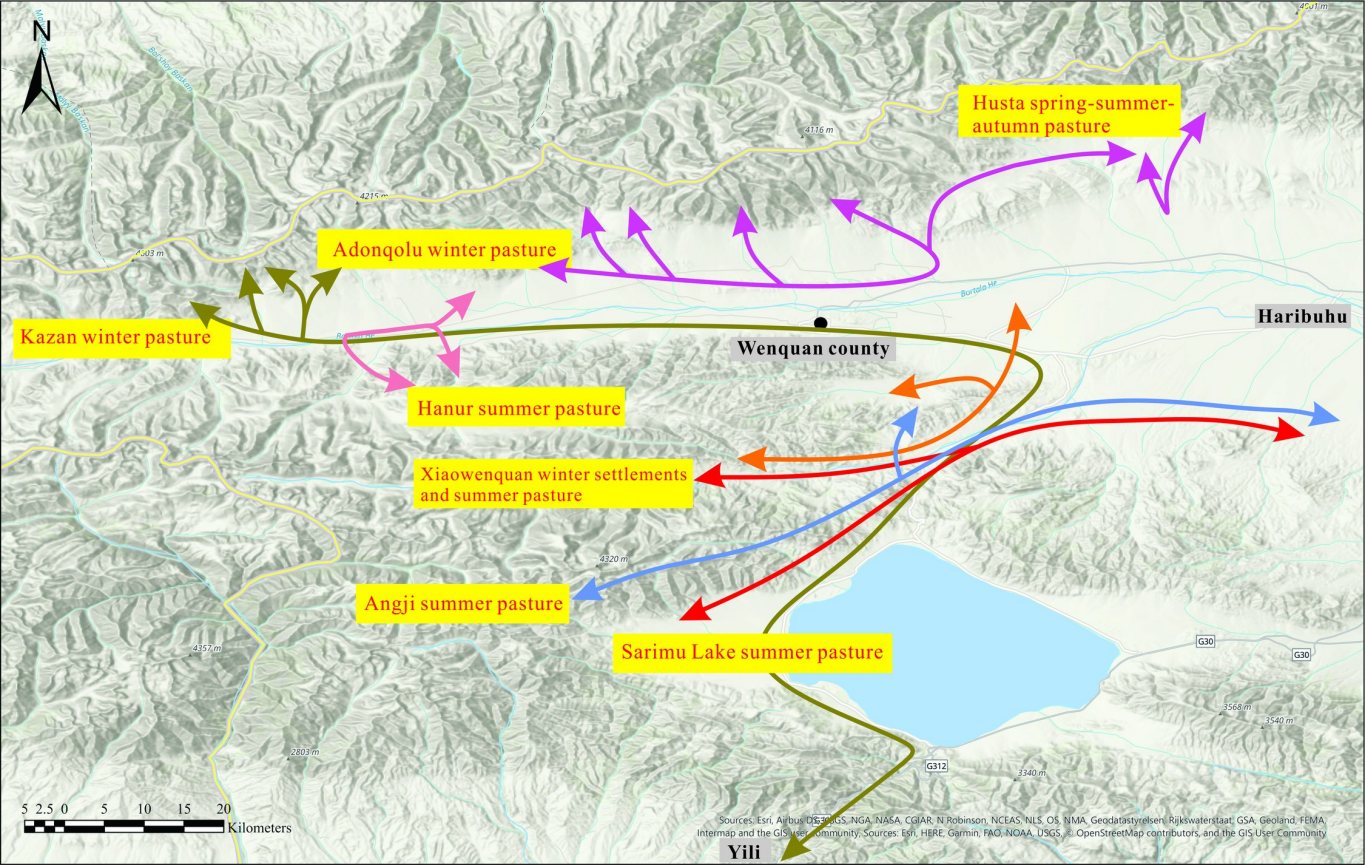
Transhumant routes among the local herders in Wenquan. Map created using ArcGIS Pro 2.5.0. Single use licence issued to Meng Qi by ESRI Australia Pty Ltd (Environmental Systems Research Institute). We acknowledge the use of Esri base maps as noted in the image.

**Fig 4:**
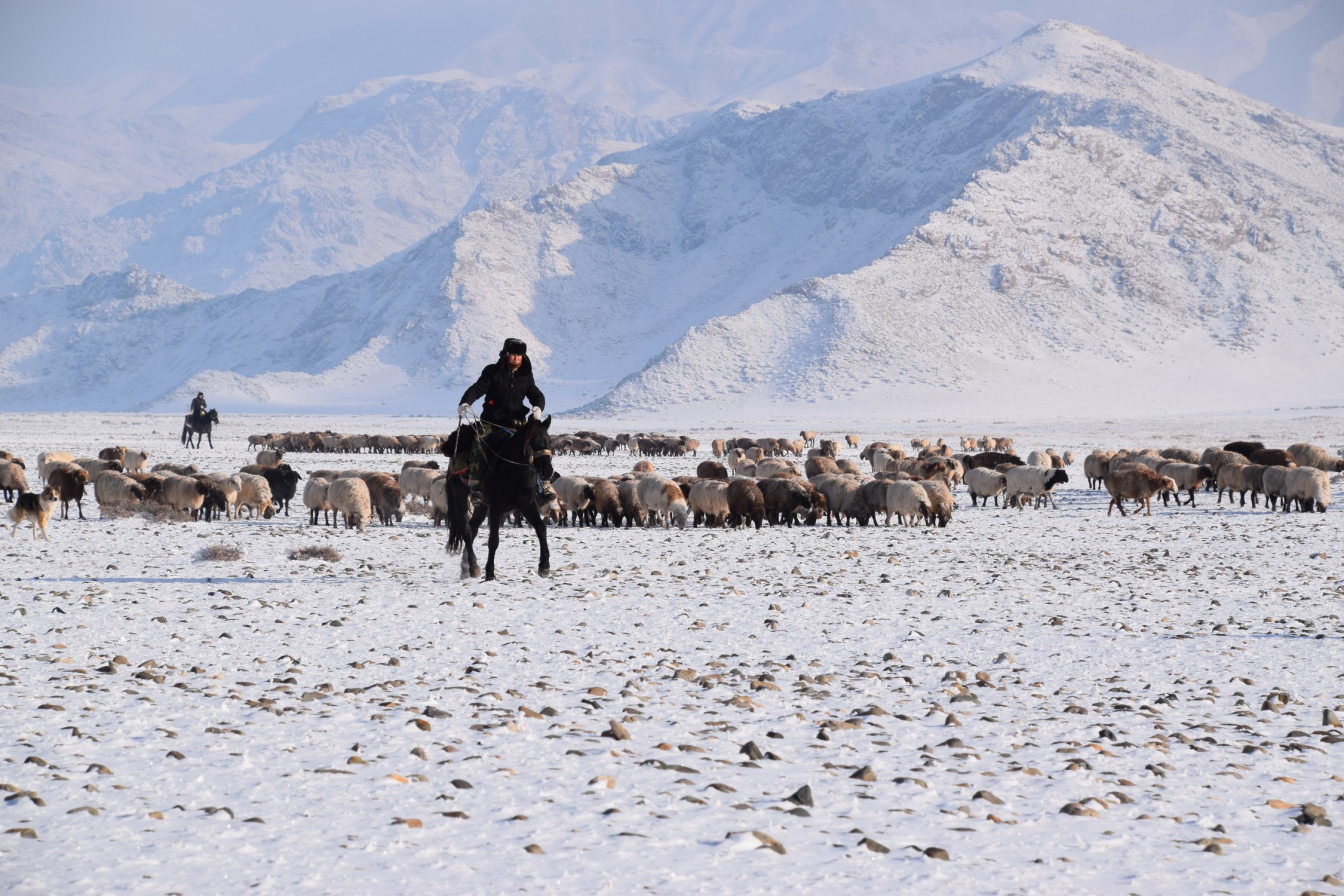
Kazak herders in the Yili Valley migrating to their winter camp at Kazan in Wenquan in mid- November 2016 (P. Jia).

**Fig 5:**
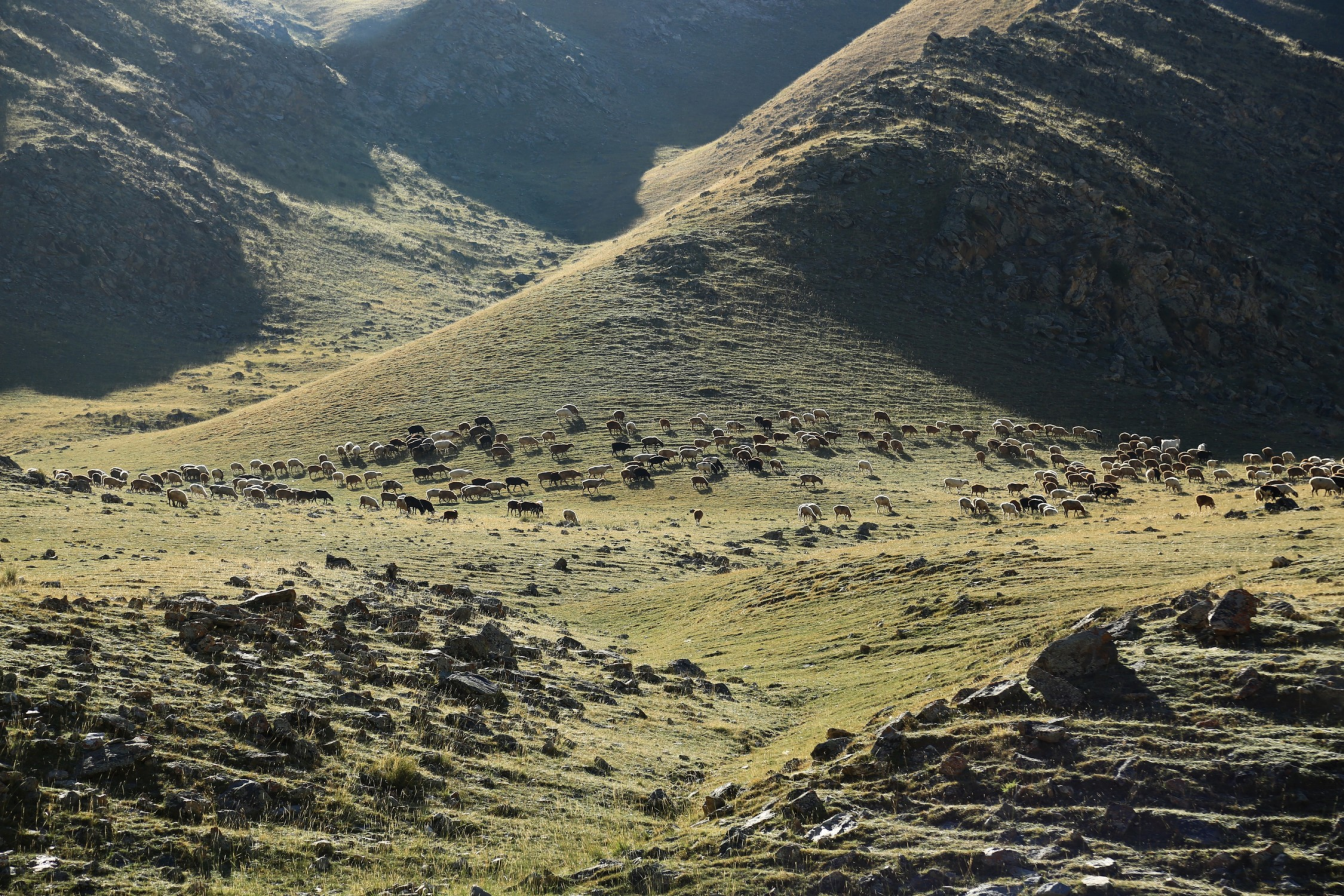
Transhumance from the summer pastures at Harnur to the autumn camp in the river valley in early September 2018 (D. Cong).

Over many generations of herding practice, local people in the Bortala Valley have developed a deep understanding of how best to use the landscape through the changing seasons (Figs 3–5). For the herders of Wenquan, the best winter pastures are in mid-altitude locations at Adunqiaolu and Kazan, while the summer pastures are located at high elevations in Husta, Harnur, Anji and around Sarimu Lake. Autumn/spring pastures are low-lying, near the river. The location of each seasonal camp is selected according to specific conditions.

Given the extremely cold climate during the winter within high latitude regions such as the Tianshan, the most important issue for local herders is to find a good location to stay during the winter. Among pastoralist societies, winter is a particularly difficult period for both humans and animals, not only due to the cold climate but also because there is a higher risk of natural disasters. Two major winter threats (*dzud*) [36, 37] are exceptionally heavy snowfall called the “white disaster”, and drought caused by very little snowfall, called the “black disaster” by herders all over the Eurasian steppe [30–33]. Heavy snowfall can cause severe problems for herders since thick snow cover can prevent the animals from accessing natural grazing. If there is insufficient fodder in storage, the animals may die in large numbers. Heavy snowfall can also cut off all road connections, leaving the herders and animals in the winter pastures isolated from major population centres. If the animals become weak and sick the herders have no access to veterinary treatment and medications. By contrast, in the case of a “black disaster”, there may be insufficient snow to provide water for both humans and animals.

**Fig 6:**
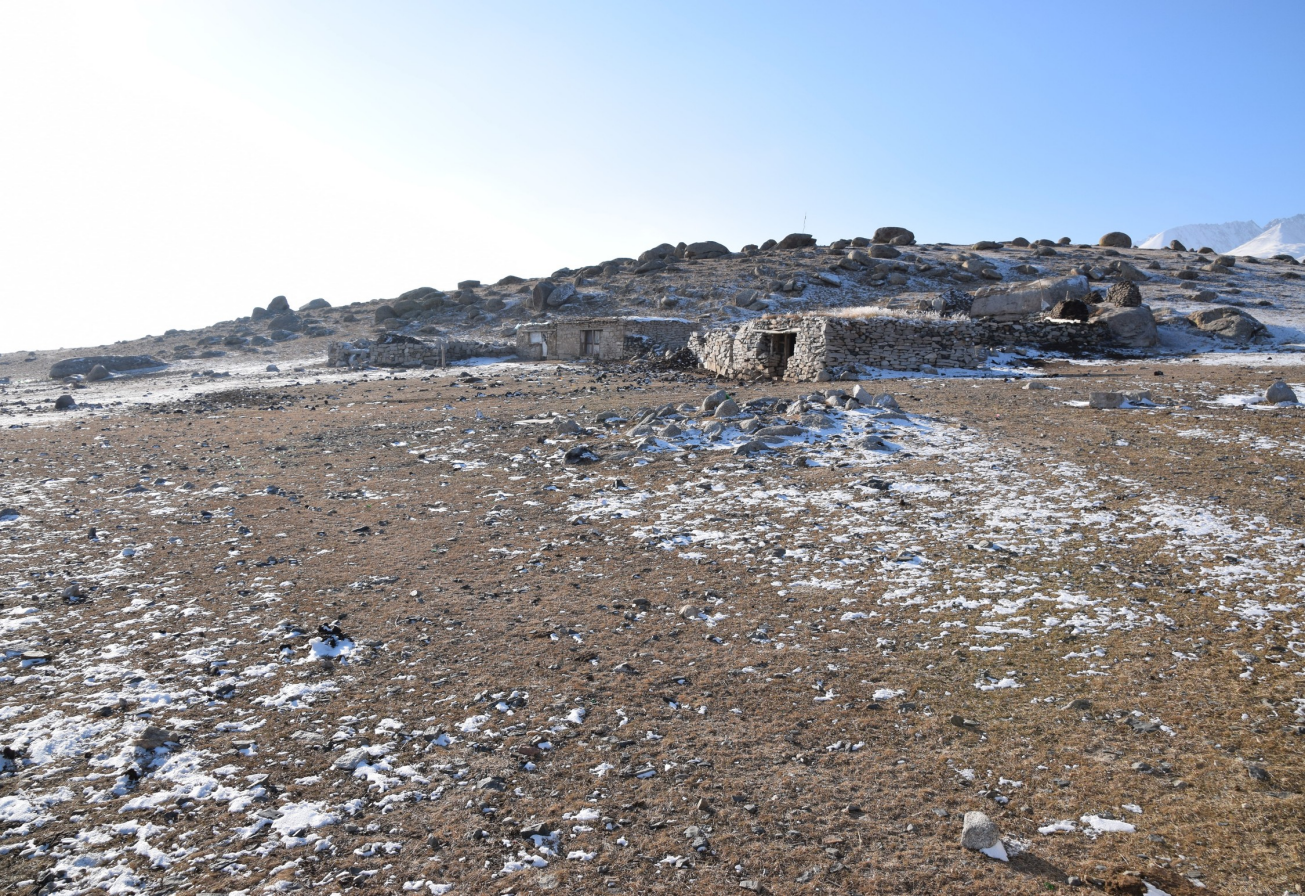
Modern winter camp at Adunqiaolu with little snow cover on the adjacent pasture. The camp faces south towards the sun with a small hill behind (P. Jia).

Over hundreds of years of experience, local herders have selected a range of different areas as optimal locations for winter pasturelands. Winter camps need to be placed in a south-facing spot sheltered by a small hill, or in a south-facing depression in a mountain valley. The shelter, and south-facing aspect exposed to maximum winter sunlight, limit the amount of snow lying on the pastureland. Sheep need less than 15 cm depth of snow accumulation to access grass under the snow. However, some snow is required so that it can be collected to provide a water supply for both humans and animals. These winter pasturelands could also be used to graze animals in other seasons as well, but this is not always the case. For instance, the grassland at Adunqiaolu about 2000 m a.s.l. is a favoured winter pastureland and although there is also good grass there in summer the local herding community conserves this by a ban on herding in this area except in winter. The Adunqiaolu pastureland is optimally suited for winter camps (Fig 6). The strong wind along the valley prevents the snow from drifting and it generally lies less than 15 cm deep, while the entire area faces south towards the sun. There are also small hills sporadically distributed across the slope, forming sheltered spots and depressions suitable for winter camps. The area has long been used in winter and today more than a hundred camps are located in this area, making it one of the best winter pasturelands in Wenquan County.

**Fig 7:**
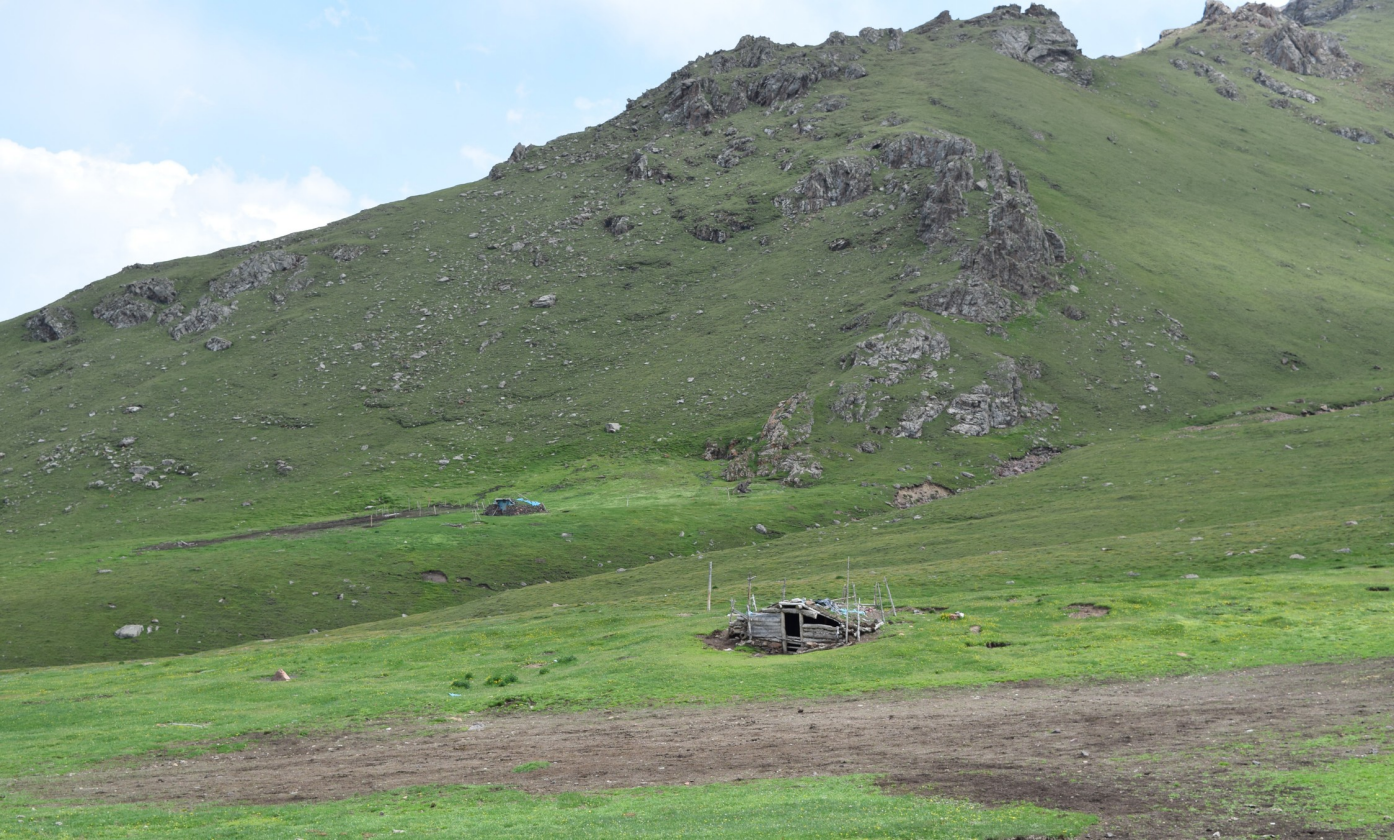
Wenquan herder Bangba’s summer camp at Big Angji, a high-altitude summer pastureland (2700 m a.s.l.) (P. Jia).

For the summer camp there is a quite different set of criteria. The site must have an open flat area to build the camp and a sheep fold. It should be cool during summer, near a fresh water supply such as a spring, a stream or a river, and there needs to be a good supply of high-quality grazing. Glacial meltwater in summer contributes to increased water flow in the rivers and streams and to the appearance of numerous springs at higher elevations [25]. In the upper Bortala Valley, these conditions for summer pastureland are usually found up in the mountain areas, up to 2600–3000 m a.s.l. in places such as Harnur, Angji and the grassland surrounding Sarimu Lake (Fig 7).

**Fig 8:**
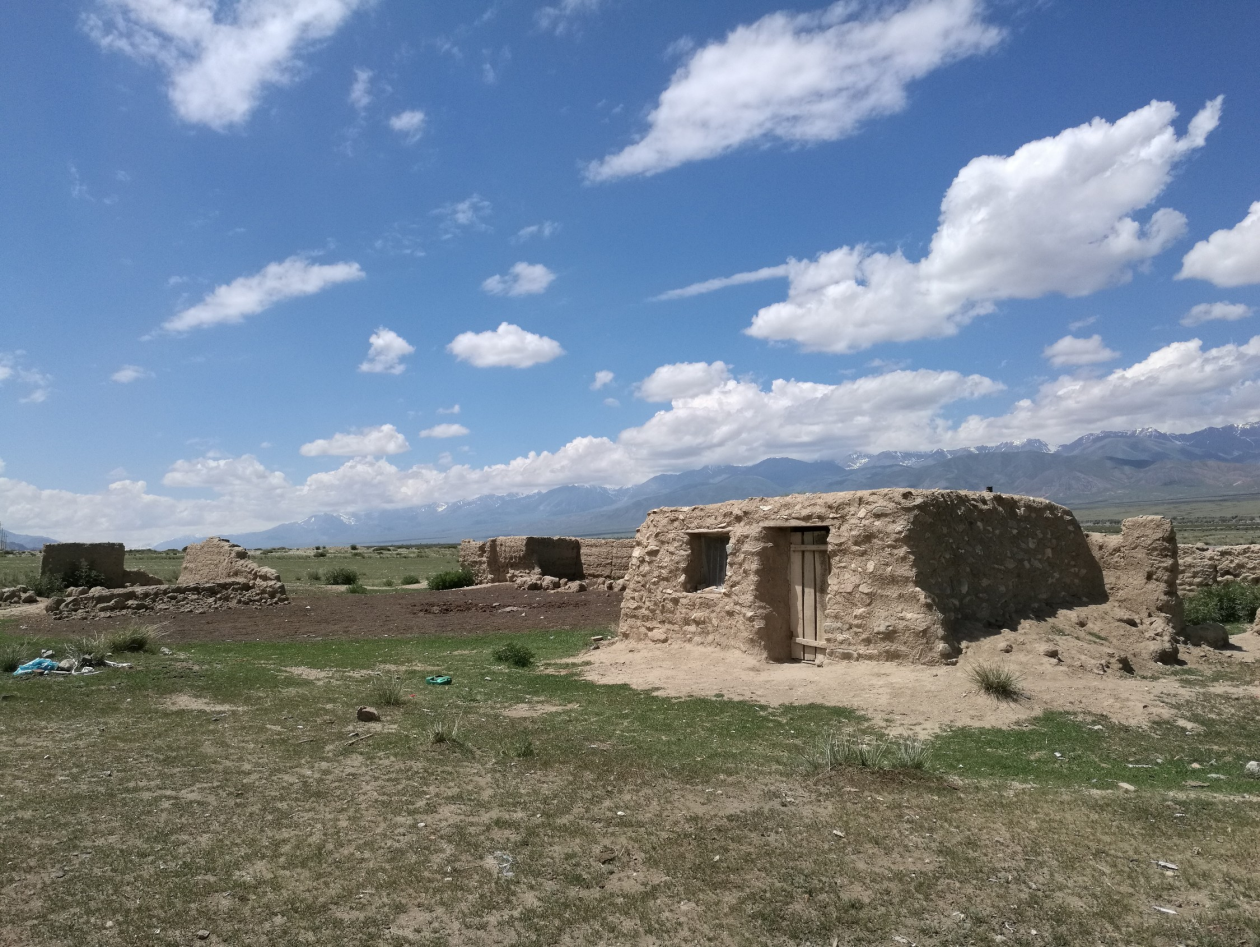
Traditional spring/autumn campsite for local herders. The site was abandoned after the herders moved to a newly constructed government funded house with an animal fold in a permanently settled village (P. Jia).

**Fig 9:**
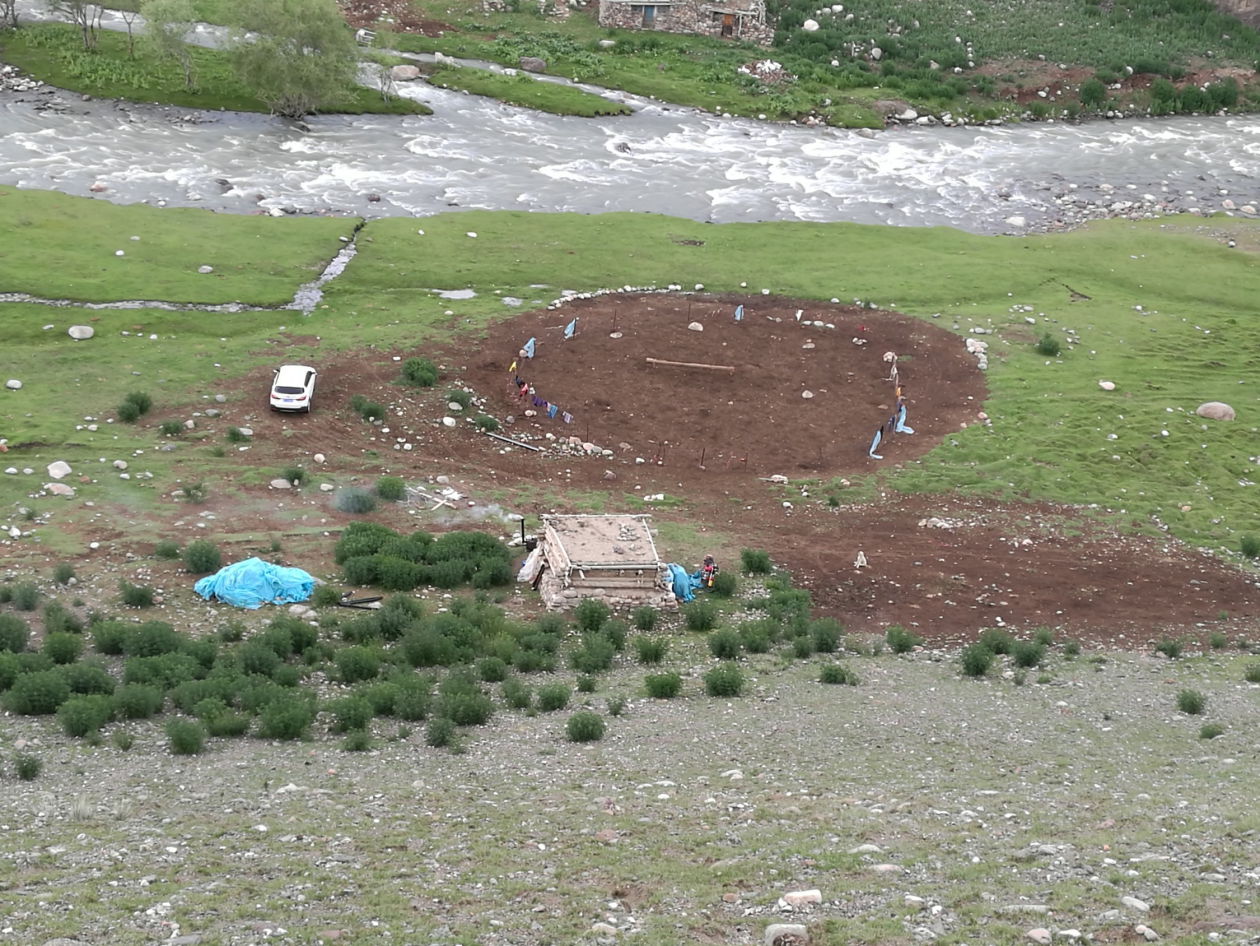
Intermediate campsite used during the spring–summer transhumance (1800 m. a.s.l.). The circular dark patch of soil surrounded by a fence is the animal fold. The small log-built hut sees only seasonal use for the herders to stay (P. Jia).

For spring and autumn, the Bortala valley pastoralists generally select a single location for both camps (Figs 8 and 9). These campsites are usually down in the river valley at a lower altitude than the winter and summer camps so that spring comes earlier, and autumn lasts longer than in the other areas. Autumn and spring are also key periods for animal reproduction, in order to time lambing to coincide with spring. Partitions are built within the sheepfolds to isolate the rams with the mature ewes in autumn and to separate out the pregnant ewes, lactating ewes and young lambs during the spring. Otherwise, rams are kept isolated from the rest of the flock for most of the year except during the winter after the ewes are already pregnant. The flat land near the river may also be used for fodder production (hay) for winter.

However, not all local herding patterns follow the “four seasons and three locations” since even within the small section of valley around Wenquan County area pasture and climate are highly diversified. For some areas such as the Husta grassland which stands at a relative low altitude (1600–1800 m a.s.l.) on the southern slope of the Alatao mountains with a moderate climate and good pasture from spring through summer into autumn [38], herders need to move only twice, once in spring up to the Husta pastureland and again in autumn to winter camps at Adunqiaolu. By contrast, some herders using relatively high altitude (2900 m a.s.l. or above) summer pastureland, such as Bangba’s (a local Mongolian herder) summer pastures, Big and Small Angji on the high slopes of the Tianshan (Fig 7), may need several short-stay intermediate campsites and pasturelands between the spring and summer pastures. Speed of movement is dictated by increasing temperatures and growth of grass during the spring. Exploiting several short-stay pastures one by one, the herders can move slowly up to the high pastures at Angji by the time full summer arrives. Such intermediate campsites are also used during the summer-autumn transhumance. When the temperature starts to fall on the high mountain pastureland in autumn, Bangba moves to the lower campsite (Fig 9), and then on again to the autumn camp when the temperature becomes too cold for the animals.

### 3.2 Ethnographic analogies

The application of ethnographic analogy to archaeological data is often perceived as problematic [e.g. 39, 40] and merits some discussion in relation to this study. In this case, there are two parts to the analogy which should be considered separately. Firstly, there are some basic constants. The landscape of the Bortala Valley has not changed substantially between the Bronze Age and the present day. Latitude and altitude remain the same and the sun still stands in the south. The needs of the domestic animals, sheep, goat, cattle and horses, while making allowances for modern breeds, remain overall very similar. There are climatic differences, as discussed below, but they are not so extensive as to radically change the types of economic options available to subsistence occupants of the valley. The second part of the analogy concerns socio-cultural patterning. Modern day herders operate in the context of an urban based market economy and within a centralised state. Bronze Age agro-pastoralists functioned as extended familial or sub-tribal groups within an unconstrained landscape. These differences influence decision making, particularly in the spring and autumn camps. The spring and autumn camps are primarily selected on the basis of early and late grazing, but today proximity to roads, transport and markets is also a factor. Access is a factor in all modern seasonal choices. Where in the Bronze Age support in times of difficulty would only have been available from near neighbours in local pastures, today regular contact is needed at all times of year between the town and the camps.

Critics of ethnographic analogies also recognise these two sides to the problem. A key issue is that cultures may diverge sharply in their responses to given economic-ecological constraints [48]. Hawkes [41] suggests that the further removed the subject of the analogy is from physical, natural constraints, the greater the ‘human’ element and thus the greater the probability of culturally diverse practices. However, we argue that in this case the degree of removal is slight. The approach taken in this paper is supported by Gould [42] who argues that if archaeologists are able to identify physical and biological limiting factors imposing essentially invariant constraints on human behaviour, it is then possible to formulate hypotheses about the behaviour of past populations under the same or very closely similar conditions. Wylie [39] notes that this relates to a very narrow set of circumstances, “in those rare limiting cases where the reconstructed behaviour is, by nature, a direct and exclusive consequence of impinging ecological or material conditions”, but she suggests that in these cases the inferences may be raised “to the level of deductive security”. Gould too agrees that such instances are rare, such as “where a particularly restrictive natural environment limits the options for survival”. We argue that the choice of seasonal encampments is, and was in the Bronze Age, governed by very specific ecological and environmental constraints which limit the influence of cultural differences to a degree that modern ethnographic data may be used to construct hypotheses about Bronze Age seasonal patterns of movement.

## 4. Methodology and results

The ethnographic study clearly shows that in understanding transhumance patterns in western Xinjiang, elevation alone is too simplistic as a proxy for seasonal patterning. Selection of seasonal locations is defined by the accessibility, quality and availability of grazing, but not necessarily all together. Winter accessibility is intricately linked to snow cover which sees major variations throughout the years. Pasture quality is related to the available water supply for a specific range land. Considering these as the main environmental parameters which define the ideal usage pattern of a given range land, we are developing a model to determine pastoralists’ choices for seasonal movements. Like every model this is a radical simplification of a complex human-environmental interaction, but we are trying to capture the essence of what makes a specific location preferable to others with regard to a localized subsistence economy. We analyze the dynamic parameters of pasture quality and snow cover throughout the year. It is clear that this approach omits for example interactions between different human groups, such as access limitations, traditionalized land tenure, or special treatment of parts of the landscape connected to grazing bans during certain times of the year. The limitations and simplicity, however, have the advantage that we can validate the model with the ethnographic accounts provided above and identify mismatches and problems for which additional explanations are necessary. We want to test whether snow cover and pasture quality are factors that allow us to determine the ideal use of a particular range land and validate the outcome based on the ethnographic data.

### 4.1 Study area spatial limits and boundary definitions

**Fig 10:**
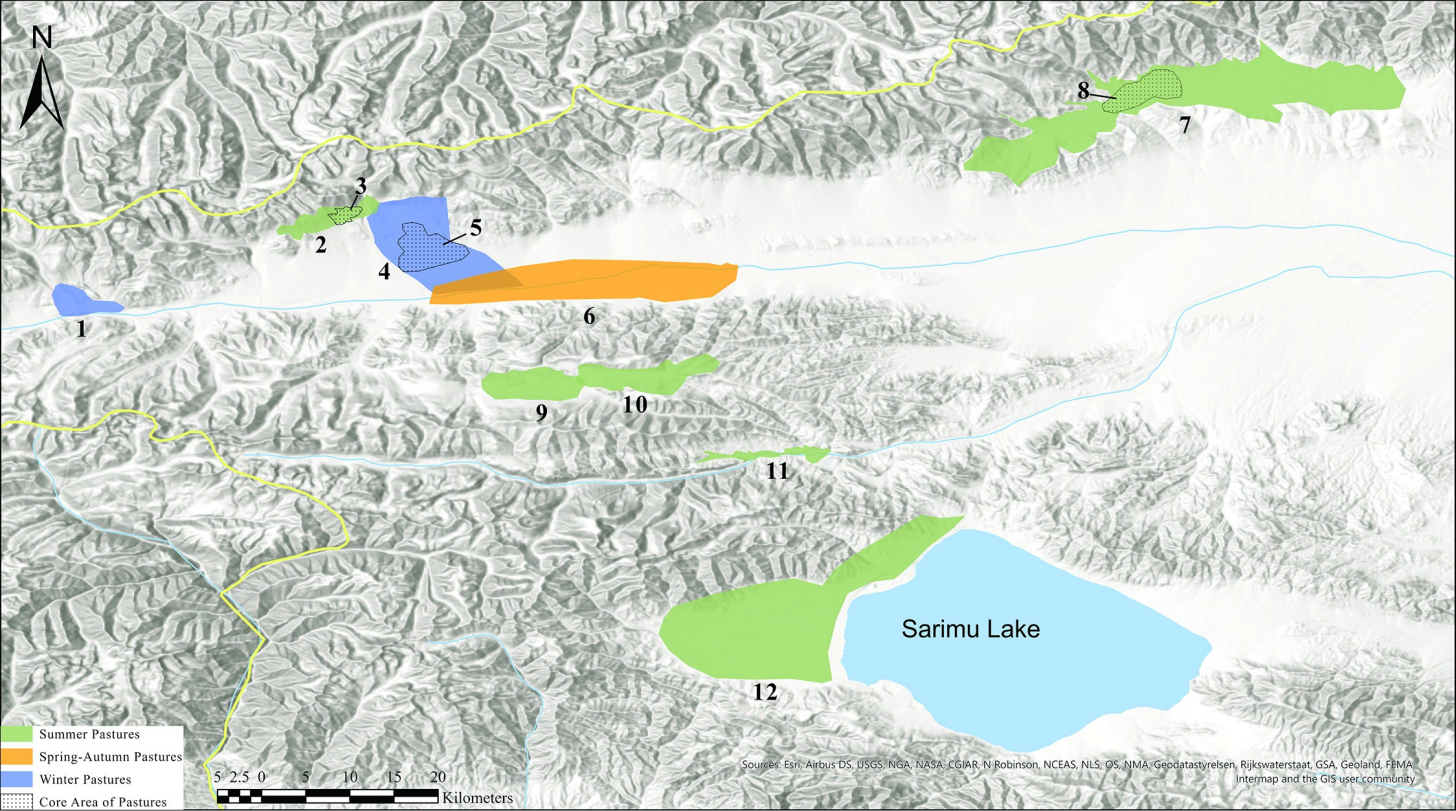
Location and area of seasonal pastureland in Wenquan: 1. Kazan Winter pasture 2, Adunqiaolu northwest summer pasture, 3. Adunqiaolu northwest core area of summer pasture, 4. Adunqiaolu winter pasture, 5. Core area Adunqiaolu winter pasture, 6. Wenquan spring-autumn pasture, 7. Entire Husta Grassland -summer-spring–autumn, 8. The Centre of Husta-summer-spring-autumn, 9. Harnur summer 1, 10. Harnur summer pasture 2, 11. Xiaowenquan Summer pasture, 12. Sarimu Lake summer pasture. Image created using ArcGIS Pro 2.5.0. Single use licence issued to Meng Qi by ESRI Australia Pty Ltd (Environmental Systems Research Institute). We acknowledge the use of Esri base maps as noted in the image.

The analysis was conducted covering the entire Bortala Valley. In order to distinguish meaningful grazing ground entities, we applied the designations made by local pastoral nomads living in the area. Using raster data covering the entire valley, we would be able to define the suitability of a grazing area on a pixel by pixel basis (depending on the resolution of the data this is 500m x 500m for MOD10A2 and 10m x 10m for Sentinel2 red and NIR bands). However, we choose to base our evaluation on the coarser, but more meaningful ethnographic usage patterns. This allows for better delineation of the grazing grounds and a direct comparability as to whether the applied approach is able to reflect the current land use patterns in the area. The analyses were conducted for the following pasture areas: Adunqiaolu winter pasture, the core area of Adunqiaolu winter pasture, the Kazan winter pasture, the Wenquan spring/autumn pasture, the Husta spring/summer/autumn pasture, the core area of the Husta pasture, the eastern and western part of the Harnur summer pasture, the north-western Adunqiaolu summer pasture, the core area of the north-western Adunqiaolu summer pasture, and the Sarimu Lake summer pasture (Fig 10). The delineation of these pasture areas is based on the ethnographic survey described above. Local herders mentioned these areas of usage to P. Jia, who then mapped the extent of the range lands by the key points derived from the handheld GPS. We analysed three areas which were designated as winter pastures through the ethnographic survey: The Adunqiaolu winter pasture, its core area, and the Kazan winter pasture. Note that the core area of the Adunqiaolu winter pasture is fully included in the larger Adunqiaolu winter pasture and therefore highly correlated with the latter. Three areas which were considered to be fit for grazing in spring and autumn were analysed: the Wenquan spring/autumn pasture, the Husta spring/summer/autumn pasture, and its core area. Five designated summer pastures were analysed: Harnur East summer pasture, Harnur West summer pasture, Adunqiaolu Northwest summer pasture and its core area, and the Sarimu Lake summer pasture. Again, Adunqiaolu and its core area are ethnographically distinguished, and the former encompasses the latter which leads to a high degree of correlation. Harnur East and Harnur West are connected.

### 4.2 NDVI processing

The Normalized Difference Vegetation Index (NDVI) is one of the most important methods for vegetation monitoring since Rouse developed it in 1972 [43]. This method is mainly dependent on the difference between healthy green vegetation and other land surface reflectance (including unhealthy/dry vegetation) in both red and infrared wavelengths [44]. The underlying principle of the NDVI is that healthy green vegetation reflects more infrared radiation and absorbs more energy in the red wavelength when compared with damaged vegetation or non-vegetated surfaces [44].

The NDVI value is defined by the ratio of:

$$NDVI=\frac{NIR-RED}{NIR+RED}$$ (1)

where NIR is the near-infrared wavelength reflectance, and RED is the reflectance in the red wavelength. The resulting values are ranging from −1 to 1, where −1 indicates no presence of vegetation at all, and 1 indicates dense levels of healthy vegetation [44].

In this model, 161 Sentinel-2 images acquired between December 2016 and February 2018 [45], were utilized to calculate NDVI rasters for the Bortala Valley, Xinjiang, China. 96 images were acquired by Sentinel-2A (S2-A) sensor between December 2016 and February 2018, and sixty-five images were acquired by Sentinel-2B (S2-B) between July 2017 and February 2018. After each image is radiometrically calibrated, the NDVI is processed. Then, a stack of NDVI results is created for each month between December 2016 and February 2018 as presented in (Table 1). We created a mosaic for each month based on the highest NDVI value among all stack images. The selection of the maximum value was chosen in order to overcome influences of cloud cover and haze problems in data acquisition. This resulted in monthly maps displaying the highest NDVI value for each pixel throughout the month. By overlaying seasonal pasture areas, the average of NDVI values within each pasture was retrieved for further analysis.

**Table 1:** Sentinel2 and MODIS data inventory.

Month	NDVI analysis data			Snow Cover Analysis
	Sentinel2-A	Sentinel2-B	Total images	MODIS (MOD10A2)
Dec. 2016	10	-	10	4
Jan. 2017	6	-	6	4
Feb. 2017	6	-	6	3
Mar. 2017	8	-	8	4
Apr. 2017	6	-	6	4
May 2017	6	-	6	4
Jun. 2017	6	-	6	3
Jul. 2017	6	6	12	4
Aug. 2017	8	6	14	4
Sep. 2017	4	5	9	4
Oct. 2017	8	6	14	3
Nov. 2017	4	10	14	4
Dec. 2017	8	12	20	4
Jan. 2018	4	12	16	4
Feb. 2018	6	8	14	3

### 4.3 Snow cover analysis

This study used Moderate Resolution Imaging Spectroradiometer (MODIS/Terra) Snow Cover data (MOD10A2) [46] for snow cover analysis during the study period. These data provide the maximum extent of snow and ice coverage within eight days [47] and use a snow-conservative approach for snow detection based on reflectance features. Data are then screened for false snow detections [48]. The MODIS snow cover data are mainly based on the Normalized Difference Snow Index (NDSI) snow-mapping algorithm. NDSI is based on the high reflectivity of snow in the visible part of the spectrum (VIS) and low reflectance in the shortwave infrared spectrum (SWIR). We can create a normalized difference index for snow similar to the NDVI [49], based on the ratio in equation number 2:

$$NDSI=\frac{VIS-SWIR}{VIS+SWIR}$$ (2)

In MOD10A2 data, snow cover was mapped by select MODIS radiance data of band 4 for VIS, and band 6 for SWIR. Snow presents a value of >0.0 in the NDSI. However, not all surfaces within these characteristics are snow which can result in a snow commission error. Especially within cloud fringes similar values can be encountered. Hence, these data must go through a process of snow commission errors alleviation. Several data screens were applied based on the spectral features of snow and other characteristics to flag uncertain snow cover detection and reverse it [48].

The MOD10A2 consists of two products; the first displays the mapped maximum snow extent over a period of eight days. The second displays the chronology of snow occurrence observations during the data acquisition period [47]. Here, we used the second product that displays snow continuity during an eight-day period as a chronology of observed snow cover. In this product, cloud cover was only included if there was persistent cloud cover on all eight days. If this was the case, then cloud cover was reported for a grid cell and the cell was not included in the analysis [48].

Table 1 presents the number of MOD10A2 data products used for each month during the study period. We classified monthly snow cover in each pasture to thin, moderate, and thick based on the average snow cover. If an area was covered in all eight-day datasets for a specific month it was considered thick coverage. When it was covered in only one out of three or four datasets, it was considered thin coverage. If the value fell between the two, we assigned moderate coverage. Finally, the percentage of each category including no snow cover was calculated to describe relative snow coverage over pastures surfaces for each month.

### 4.4 Results

Below we go through each area designated by local pastoralists as having a specific usage, briefly elaborating on the vegetation growth cycles and the snow fall patterns as well as any issues with our simplified modelling approach. We group these areas into the seasonal movement stages they belong to in order to see whether they do show commonalities or disparities.

#### 4.4.1 Winter grazing grounds

As expected, the main requirement for a winter pasture is limited snow cover during the cold months of the year, so animals can access food. In all three areas we see little snow in the months of December through March. For Adunqiaolu there is some thicker snow cover in February 2017, but then the next year sees almost no snow in the same month. Kazan shows snow in December of 2016 but is completely free of snow in December 2017. Crucially, there is no month during the year which sees complete thick or moderate snow cover. Some parts of the pasture always remain open or are at most temporarily covered with a thin layer of snow. Clearly the significance of observations made during two consecutive years is limited, but neither of the years was considered extreme by the locals. Neither black nor white disasters were recorded for the time period the underlying data of the model date to (December 2016 –February 2018). Hence, the time period covered can be assumed to lie within the range of normal years where no shocks to the pastoralist system and therefore no changes in herding patterns are observed.

The limited snow cover during the winter months is connected to the low quality of the pasture in the following months. Even during the peak of the vegetation cycle in July the average NDVI value barely exceeds 0.3 for the Adunqiaolu winter pasture and its core area and 0.25 for the Kazan winter pasture. The grazing grounds dry off quickly in summer and the NDVI values decline. It is important to note that the NDVI values for barren areas, rock, and snow lie around 0.1 to -0.1. Considerable snow cover therefore impacts the average NDVI values and these averages are not directly connected to the quality of the grazing during the winter months. Slightly higher grazing qualities can be expected since snow cover biases the NDVI of a pasture area towards 0. Average NDVI values for winter pastures during the months of usage seem to hover around a value of 0.1.

#### 4.4.2 Spring/autumn grazing grounds

Spring/autumn pastures are more difficult to identify, since they serve a transitional function and are usually only used during six to eight weeks from late March to early May and from late September to early December. In terms of snow cover the data showed a still considerable snow cover in March. With the beginning of April, the vegetation saw rapid development which is consistent with the first movement of pastoralists during the year. However, both Husta, including its core area, as well as the Wenquan spring/autumn pasture showed characteristics that need to be further elaborated upon since they demonstrate the limits of a pasture classification approach based on NDVI averages and snow cover. Wenquan, whereas consistent with the melting period and the first move of the year, showed very low NDVI averages over the entire year, even trailing the overgrazed and dried off winter pastures. The topographic location of the Wenquan spring/autumn pasture offered explanations beyond the simplistic assumptions of the model.

The Wenquan pasture seemed to only provide meagre grazing throughout the entire year. The area lies to both sides of a river. Water has a very low NDVI value approaching -1 and could considerably influence an average. However, even when excluding the river and the denuded surfaces of the riverbed, the NDVI average stayed low. Therefore, other explanations needed to be considered. According to ethnographic data, the most important reason for the usage of Wenquan as a spring pasture was the early temperature increase which allows for a shortening of the winter camping time. These range lands provided a convenient location within the landscape as a transition area for heading up to the summer pastures as soon as snow started to melt there. It also had small areas of lush vegetation next to the riverbank which were too small in area size to influence the overall NDVI average much but provided ideal grazing for a limited amount of time. The abundant availability of water next to the river was convenient for herders and even allowed for limited agricultural activity including fodder crops and sometimes vegetables. Beyond environmental factors, Wenquan served as a place for exchange of animals and goods and stacking supplies for winter/summer periods.

The Husta grazing lands are not only used in spring and autumn but also in summer. The reason for this lies in the favourable topographic location of the pasture. Its lower elevation starts around 1400 m a.s.l. and the highest areas lie at 2200 m a.s.l. This allows for slow movements inside the pasture area itself, depending on where the grazing is best. To the south, the Husta pasture is framed by a small ridge of around 1600–1900 m a.s.l. The north side is backed by the mountain range which defines the Bortala valley [38]. This creates a topographic situation where seasonal springs and small creeks provide water for the vegetation throughout the growing season from spring to autumn and create one of the best grazing grounds in the entire valley.

#### 4.4.3 Summer grazing grounds

The summer pastures are characterized by late snow melt in the spring, usually in the month of April, with a good chance of considerable quantities of snow by October. Harnur East and West, as well as the summer pasture west of the Sarimu Lake, clearly fit this pattern. Harnur East, one of the highest lying summer pastures, shows consistent snow cover of between 70% and 100% from October through April, with most areas displaying thick or moderate snow cover. The Adunqiaolu Northwest summer pasture and its core area can experience full snow cover during the winter months (December 2016), but otherwise see relatively little snow during the study period. We think that the chance of a full moderate or thick snow cover excludes this area from safe usage during the winter, but there may be sociocultural and practical factors at play as well. As discussed earlier, less snow cover is not the only condition to be considered in defining a suitable winter pastureland. Winter camping grounds also require specific landforms such as a small hill or depression for shelter as well as easy external access. At an elevation of 2900 m a.s.l. the Adunqiaolu Northwest summer pasture is extremely cold in winter and the landforms offer no places for good shelter from the wind. The track leading up to the pastures is difficult, particularly in winter when it would be necessary to climb or descend steep, potentially snow-covered slopes.

All summer pastures show a late but accelerated start to vegetation growth in the month of May. This fits with the second movement of the year in early May. The herds arrive on the summer pastures around the time when vegetation growth is spiking up from its low level in April. The average NDVI values for the summer pastures peaks in July with values around 0.7. These average values are extremely high for grassland and speak for the excellent quality of the grazing on these summer pastures. NDVI has been shown to correlate with productivity [50, 51]. The interrelations are certainly more complex, but as an approximation the summer pastures can be seen as roughly twice as productive as the winter pastures in our case. This has clear implications for the calorie intake of the animals over the course of the year and demonstrates why these seasonal movements are worthwhile.

## 4.5 Summary

**Fig 11:**
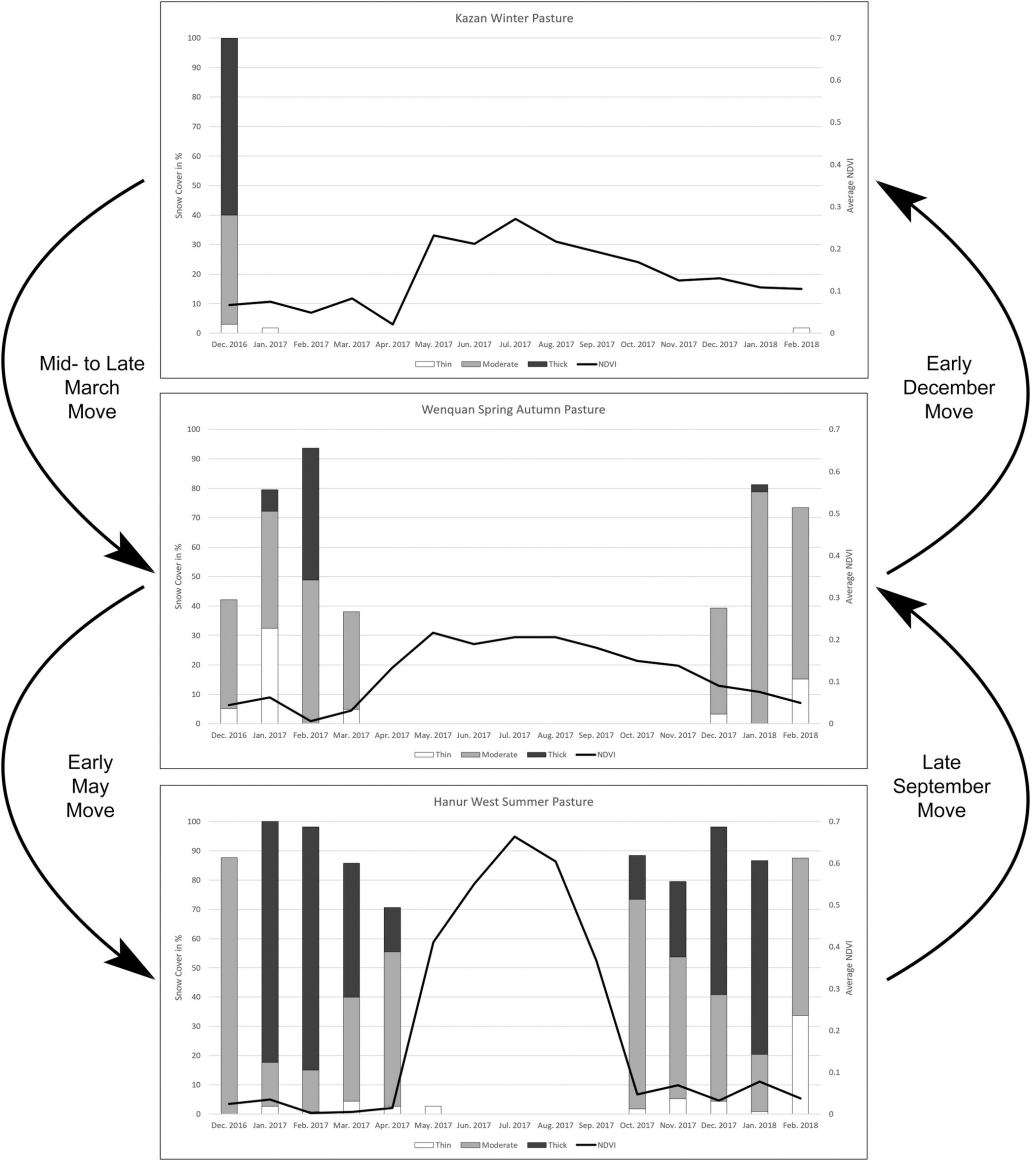
Idealised pastoralist movements based on ethnographic and remote sensing data. Left y-axis: snow cover in % displayed as a bar diagram distinguishing thin/moderate/thick snow cover. Right y-axis: average NDVI value.

The general model does provide insights into potential usage patterns of the landscape (Fig 11). The combination of snow cover and grazing quality seems to show relatively clearly what a particular area is best used for and the results match up with the ethnographically assigned usage. The Kazan winter pasture, for example, has relatively low pasture quality, but it is almost free of snow in the harshest winter months of January and February, thus guaranteeing access of animals to food. On the Wenquan spring pasture, the growth phase starts early and when the first seasonal movements are made by pastoralists, the pasture is already relatively lush, seeing additional growth throughout the months of April and May. The quality of the grass then stays fairly consistent throughout the summer. The herds, however, are moved to the summer pastures like, for example, Sarimu. These have been under thick snow all winter, but as soon as it melts in late April / early May, the intensive growth phase starts. These pastures provide the best grazing throughout the summer into early fall. Before the first snow starts covering the ground in October, the animals are moved back to the autumn pasture where they can graze on the grass that was left untouched throughout the summer for a few more weeks before eventually the move to the winter pasture completes the year.

## 5. Discussion

Studying patterns of modern seasonal herding movement, reinforced by analysis of pasture quality and snow cover, makes it possible to infer specific seasonal occupation periods for Bronze Age house sites. The archaeological evidence for Bronze Age occupation sites is limited but sufficient to begin to address this question in a meaningful way.

### 5.1 Archaeological site distribution as compared to model-identified pastures

#### 5.1.1 Winter pastures

Terrain analyses were conducted for the wider Adunqiaolu winter pasture as well as the core area, and also the Kazan winter pasture. Adunqiaolu, as the area studied most intensively, has the strongest evidence for seasonality of use. Archaeological field survey and excavation has recorded more than a hundred campsites around Adunqiaolu so it is clear that the location was a favoured one in the Bronze Age. Several factors suggest that House F1 at Adunqiaolu [18] was a winter encampment. A modern winter camp is located immediately adjacent to F1, both taking advantage of the shelter provided by a low hillock just behind the houses (Fig 6). Local informants testify that this pasture is ideal for, and in fact specifically reserved for, winter camps, while the analysis of snow cover confirms these ethnographic accounts. Botanical studies provide additional supporting evidence. Pollen analysis of sheep dung from the Bronze Age house exhibits a much more limited variety of species than modern sheep dung collected near there in summer (sheep belonging to the pasture guardian protecting the lands from out of season use), while the phytoliths present in the modern summer sheep dung come from less mature plants than those in the Bronze Age dung, suggesting that the animals were very likely kept in the house during autumn or winter [52].

#### 5.1.2 Summer pastures

Terrain analyses were conducted for Husta spring/summer/autumn pasture, the core area of the Husta pasture, the eastern and western part of the Harnur summer pasture, the north-western Adunqiaolu summer pasture, the core area of the north-western Adunqiaolu summer pasture, and the Sarimu Lake summer pasture. Husta is a particularly favoured pasture, available from spring through summer into autumn. There is archaeological evidence to suggest that it was so significant in the Bronze Age that it was necessary to defend it with walled lookout posts [29]. More than a hundred Bronze Age campsites have been identified there. Harnur is a smaller pasture and the season of possible use is much shorter than at Husta. Harnur East is one of the highest lying summer pastures. Fewer than ten campsites have been recorded there across both Harnur East and West, but this is still sufficient to demonstrate its use in the Bronze Age. There are also high mountain summer pastures at Adunqiaolu where more than twenty camps have been identified. No survey has been conducted in the Sarimu Lake summer pasture.

#### 5.1.3 Spring/autumn

Terrain analyses were conducted for the Wenquan spring/autumn pasture and the Husta spring/summer/autumn pasture. The low-lying land around Wenquan has been subject to considerable modern disturbance and it has proved difficult to undertake survey there, so the presence of Bronze Age sites cannot yet be confirmed. However, the terrain analysis matches the ethnographic accounts, showing that the early appearance of grazing and its late persistence is more important in spring and autumn than the quality, as it helps to protect use of the winter pastures, the most critical of all throughout the seasons.

### 5.2 Paleoclimate

For the period of maximum Bronze Age occupation in the Bortala Valley from c. 3900–3400 cal yr BP [18] available palaeobotanical data indicate steppic vegetation broadly similar to that of the present day. However, there are shifts within the paleoclimate that may have impacted Bronze Age land use. Holocene climate records for western Xinjiang and surrounding regions indicate localised variation over time, but they do exhibit a general pattern [53–55]. The start of the Holocene saw generally arid vegetation in low lying areas, and steppe and forest at high elevations. This changed with a somewhat cooler, wetter period starting around 6400 to 5500 cal yr BP, lasting until c. 4000 cal yr BP when there is a warming shift again. Cooler and wetter conditions return in the very late Holocene. Within the Bortala valley itself, a recent study of sediment from the Wenquan wetlands showed a shift from an arid sub-desertic landscape to steppic conditions around 7500 cal. yr BP, with a more humid steppic environment from c. 4200 cal. yr BP (J. Dodson, pers. comm.). The Andronovo culture in Xinjiang is dated between the 18^th^ and the 14^th^ centuries BCE, coinciding with the post 4000 BP warming shift. This may have created more favorable conditions for summer grazing and may also have ameliorated the severity of winter snowfall. Generalized as this evidence is, it indicates suitable conditions for Bronze Age transhumant pastoralism and does not contradict the results of the combined archaeological, ethnographic and terrain analyses discussed above. As with the ethnographic data, for considerations of the influence of climate on site patterning, some constants such as elevation, aspect and access still remain. The presence of residential structures in the Adunqiaolu winter pastures, with botanical evidence supporting their use in winter, suggests that snow cover was also slight there in the Bronze Age. The large number of residential structures at Husta [29] indicate that this was also a very much favored summer pasture in the Bronze Age, although it is not clear as to how early and how late in the season it was used.

### 5.3 Pastoral mobility

Despite the apparent domestication of the horse at Early Bronze Age Botai in northern Kazakhstan [56], horses did not form a significant component of Middle and Late Bronze Age pastoral economies in the eastern Eurasian steppe which were dependent on cattle, sheep and goat, in varying proportions determined by the micro-niches they exploited. Studies at Begash in the western Dzhungar Mountains show a focus on sheep and goat over cattle as early as the mid-3^rd^ millennium BCE [25]. This may have helped to encourage the development of the vertical pastoralism that facilitated the spread of Andronovo groups in and across much of Xinjiang during the 2^nd^ millennium BCE. This pattern of a predominance of sheep and goat, with fewer cattle, and horses exploited mainly for transport, is also present today in western Xinjiang. However, from the Iron Age up to Late Medieval times, the steppes were dominated by mounted tribal confederacies whose wide-ranging movements were facilitated by their reliance on the horse for food, transport and warfare. This shift to new economic practices and subsequent increasing social complexity can be seen by the Late Bronze Age with the appearance of the Deer-Stone Khirigsuur culture [57, 58] and the emergence of highly mobile nomadic pastoralism associated with the earliest “Scythian” horizon [59–62]. In western Xinjiang, traces of the Andronovo fade away towards the end of the 2^nd^ millennium BCE, to be replaced by transitional Bronze/Iron Age groups who may have exploited the landscape in quite different ways. Over the past few centuries, with increasing state control over the steppes, and over Xinjiang, tribal confederacies gradually lost their power. Together with wider access to market economies, lifestyles began to differentiate again according to local conditions, permitting us to draw close parallels between the Bronze Age and the present day.

## 6. Conclusion

While it has been generally accepted that the Eurasian Andronovo peoples were mobile pastoralists, details of their specific adaptation to transhumant pastoralism in the Inner Asian Mountain Corridor have not been widely explored archaeologically. This study, using detailed ethnographic fieldwork and analysis of modern snow and grass cover, has shown that there is a strong correlation between modern patterns of seasonal movement and those apparently practiced in the Bronze Age. Investment in built structures in both periods further reinforces the close relationship between the two. Botanical data obtained from House F1 in the Adunqiaolu winter pastures provides a range of evidence strongly supporting the hypothesis that the structure was used as a winter camp while the widespread distribution of Bronze Age sites in modern winter and summer pastures emphasises similarities in patterns of seasonal movement between the two periods.

## References

[1] Stenning D. J. Transhumance, migratory drift, migration; patterns of pastoral Fulani nomadism. The Journal of the Royal Anthropological Institute of Great Britain and Ireland 1957; 87(1): 57–73.

[2] Frachetti M. D. Variability and Dynamic Landscapes of Mobile Pastoralism in Ethnography and Prehistory. In: Barnard H, Wendrich W, editors. The Archaeology of Mobility: Nomads in the Old and in the New World, Los Angeles: Cotsen Institute of Archaeology UCLA; 2008 pp. 366–396.

[3] Allentoft M. E., Sikora M., Sjögren K. G., Rasmussen S., Rasmussen M., Stenderup J., et al Population genomics of bronze age Eurasia. Nature 2015; 522(7555): 167–172. 10.1038/nature14507 26062507

[4] Wilkin S., Miller A. V., Taylor W. T., Miller B. K., Hagan R. W., Bleasdale M., et al Dairy pastoralism sustained eastern Eurasian steppe populations for 5,000 years. Nature ecology & evolution 2020; 4(3): 346–355. 10.1038/s41559-020-1120-y 32127685 PMC7212056

[5] Yu J. New discoveries of the excavation during 2016–2017 at the Tongtiandong site, Jimunai County in Xinjiang. The Western Regions Studies, 1:132–135.于建军. (2018). 《2016 ~ 2017 年新疆吉木乃县通天洞遗址考古发掘新发现》. 《西域研究》(1), 132–135.

[6] Liu H, Te’erbayi’er Wang X, Guanba Liu H. Preliminary analysis of rescue excavation on the road construction at the Nileke section of Dunna Freeway in Yili, Xinjiang. The Western Regions Studies, 3:137–139. 刘汉兴, 特尔巴依尔, 王晓丹, 关巴, & 刘慧娟. (2018). 《新疆伊犁州墩那高速尼勒克段考古收获及初步认识》. 《西域研究》(3), 137–139.

[7] XIA. Xinjiang Institute of Archaeology and Relics. Brief excavation report on the Ayituohan I cemetery in Habahe County. Xinjiang Relics 2017;2:19–39. 新疆文物考古研究所. (2017). 《哈巴河县阿依托汗一号墓群考古发掘报告》. 《新疆文物》(2), 19–39.

[8] Jia P, Betts A. A re-analysis of the Qiemu’erqieke (Shamirshak) cemeteries, Xinjiang, China. Journal of Indo-European Studies 2010;38(3–4): 1–43.

[9] Abuduresule Y, Li W, Hu X. The Xiaohe (Small River) Cemetery and the Xiaohe Culture. In: Betts A, Vicziany M, Jia P, Di Castro AA, editors. The Cultures of Ancient Xinjiang, Western China: Crossroads of the Silk Roads. Oxford: Archaeopress; 2019 pp. 19–51.

[10] Betts A., Jia P., & Abuduresule I. A new hypothesis for early Bronze Age cultural diversity in Xinjiang, China. Archaeological Research in Asia 2019: 17; 204–213.

[11] Kovalev A.A. Earliest Europeans in the Heart of Asia: The Chemurchek Cultural Phenomenon. St. Petersburg: St. Petersburg State Museum; 2015.

[12] Li C., Li H., Cui Y., Xie C., Cai D., Li W., et al Evidence that a West-East admixed population lived in the Tarim Basin as early as the early Bronze Age. BMC Biology 2010; 8: 15 10.1186/1741-7007-8-15 20163704 PMC2838831

[13] Li C., Ning C., Hagelberg E., Li H., Zhao Y., Li W., et al Analysis of ancient human mitochondrial DNA from the Xiaohe cemetery: insights into prehistoric population movements in the Tarim Basin, China. BMC Genetics 2015; 16:78 10.1186/s12863-015-0237-5 26153446 PMC4495690

[14] Gao SZ, Zhang Y, Wei D, Li HJ, Zhao YB, Cui YQ, et al Ancient DNA reveals a migration of the ancient Di‐qiang populations into Xinjiang as early as the early Bronze Age. American journal of physical anthropology 2015;157(1): 71–80. 10.1002/ajpa.22690 25546319

[15] Koryakova L, Epimakhov A. The Urals and Western Siberia in the Bronze and Iron Age. Ser. World Archaeology; 2007.

[16] Kuz'mina E. The origins of the Indo-Iranians. Leiden: Brill; 2007.

[17] Kuz'mina E. The prehistory of the Silk Road. Philadelphia: University of Pennsylvania Press; 2008.

[18] Jia P, Betts A, Cong D, Jia X, Dupuy P. Adunqiaolu: new evidence for the Andronovo in Xinjiang, China. Antiquity 2017;91(357): 621–639.

[19] Cong D. Tianshan as a Bridge: New Studies of Bronze Age Archaeology in the Western Tianshan, Xinjiang, China. In: Betts A, Vicziany M, Jia P, Di Castro AA, editors. The Cultures of Ancient Xinjiang, Western China: Crossroads of the Silk Roads. Oxford: Archaeopress; 2019 pp. 52–63.

[20] Natural Geography of Wenquan County adapted on June 5, 2020 from Wenquan County Council Official Website. Available from: http://www.xjwq.gov.cn/info/1022/1024.htm.

[21] National Meteorological Science Data Centre, adopted on June 5, 2020. Available from: http://data.cma.cn/dataService/cdcindex/datacode/A.0029.0003/show_value/normal.html.

[22] Zhang X. Vegetation map of China and its geographic pattern: illustration of the vegetation map of the People’s Republic of China (1:1000,000). Beijing: Geological Publishing House; 2007.

[23] Caspari G, Plets G, Balz T, Fu B. Landscape archaeology in the Chinese Altai Mountains–Survey of the Heiliutan Basin. Archaeological Research in Asia 2017;10: 48–53.

[24] Caspari G, Quantifying the Funerary Ritual Activity of the Late Prehistoric Southern Kanas Region (Xinjiang, China). Asian Perspect. 2020;59(2): forthcoming.

[25] Frachetti M. D. Pastoralist landscapes and social interaction in Bronze Age Eurasia. University of California Press; 2009 10.1097/MED.0b013e32832912e7

[26] Caspari G. “Virtual dwellings” or architecture for the living? A hypothesis. Archaeological Research in Asia 2019 10.1016/j.ara.2019.100157.

[27] Bourgeois J, De Langhe K, Ebel AV, Dvornikov EP, Konstantinov N, Gheyle W. Geometric stone settings in the Yustyd Valley and its surroundings (Altai Mountains, Russia): Bronze Age ‘virtual dwellings’ and associated structures. Archaeological Research in Asia, 2017;10: 17–31.

[28] Jacobson-Tepfer E. The hunter, the stag, and the mother of animals: image, monument, and landscape in ancient North Asia. Oxford: Oxford University Press; 2015.

[29] Jia P, Betts A, Doumani P, Cong D, Jia X. Bronze Age Hill Forts: new evidence for defensive sites in the western Tian Shan, China. Archaeological Research in Asia 2017;15: 70–81.

[30] Khazanov AM. Nomads and the outside world. Cambridge: Cambridge University Press; 1984.

[31] Chang C. Pastoral transhumance in the south Balkan as a social ideology: ethnoarchaeological research in northern Greece. American Anthropologist 1993;95: 687–703.

[32] Arnold ER, Greenfield HJ. The origins of transhumant pastoralism in temperate southeastern Europe—a zooarchaeological perspective from central Balkans. Oxford: Archaeopress; 2006.

[33] Cribb R. Nomads in archaeology. Cambridge: Cambridge University Press; 1991.

[34] Edelberg L. Seasonal dwellings of farmers in North West Luristan. Folk 1966–67;9: 374–401.

[35] Chen X. Nomadic herders in Altai mountians -ecological environment and indigenous knowlede. Beijing: Social Sciences Academic Press; 2017;142–151. 陈祥军. (2017). 《阿尔泰山游牧者-生态环境与本土知识》. 北京: 社会科学文献出版社, 142–151页.

[36] Conte T. The effects of China's grassland contract policy on Mongolian herders' attitudes towards grassland management in northeastern Inner Mongolia. Journal of Political Ecology 2015;22: 79–97.

[37] Begzsuren S., Ellis J.E., Ojima D.S., Coughenour M.B., Chuluun T. Livestock responses to droughts and severe winter weather in the Gobi Three Beauty National Park, Mongolia. Journal of Arid Environments 2004:59(4); 785–796.

[38] Caspari G, Betts A, Jia P. The Bronze Age in the Western Tianshan, China: A new model for determining seasonal use of sites. Journal of Archaeological Science: Reports 2017;14: 12–20.

[39] Wylie A. The Reaction against Analogy. Advances in Archaeological Method and Theory 1985; 8: 63–111.

[40] Currie A. Ethnographic Analogy, the Comparative Method, and Archaeological Special Pleading. Stud Hist Philos Sci 2016 2;55:84–94. 10.1016/j.shpsa.2015.08.010 26774072

[41] Hawkes C. Archeological theory and method: Some suggestions from the Old World. American Anthropologist 1954; 56:155–168.

[42] Gould RA. Living archaeology. Cambridge:Cambridge University Press; 1980.

[43] Rouse JW. Monitoring the vernal advancement and retrogradation (greenwave effect) of natural vegetation. Nasa/gsfct Type Report; 1972.

[44] Rhew IC, Vander SA, Kearney A, Smith NL, Dunbar MD. Validation of the normalized difference vegetation index as a measure of neighborhood greenness. Annals of Epidemiology 2011;21(12): 946–952. 10.1016/j.annepidem.2011.09.001 21982129 PMC3225119

[45] Copernicus. Sentinel-2 2016–2018 data processed by ESA. 2018. Available from: http://earth.esa.int.

[46] Hall DK, Riggs GA. 2016. MODIS/Terra Snow Cover 8-Day L3 Global 500m Grid, Version 6. [Indicate subset used]. Boulder, Colorado USA. NASA National Snow and Ice Data Center Distributed Active Archive Center. 10.5067/MODIS/MOD10A2.006. [20 March 2018].

[47] MODIS. MOD10A2 / MYD10A2 snow products. 2018. Available from: https://modis-snow-ice.gsfc.nasa.gov/?c=MOD10A2.

[48] Riggs GA, Hall DK. MODIS Snow Products Collection 6 User Guide. 2016. Available from: https://nsidc.org/sites/nsidc.org/files/files/MODIS-snow-user-guide-C6.pdf.

[49] Hall DK, Riggs GA, Salomonson VV. Development of methods for mapping global snow cover using moderate resolution imaging spectroradiometer data. Remote Sensing of Environment 1995;54(2): 127–140.

[50] Paruelo JM, Oesterheld M, Di Bella CM, Arzadum M, Lafontaine J, Cahuepé M, et al Estimation of primary production of subhumid rangelands from remote sensing data. Applied Vegetation Science 2000;3(2): 189–195.

[51] Paruelo JM, Epstein HE, Lauenroth WK, Burke IC. ANPP estimates from NDVI for the central grassland region of the United States. Ecology 1997;78(3): 953–958.

[52] Shao K, Zhang J, Cong D, Jia W, Cui A, Wu N. Analysis of plant microfossils reveals the ancient survival strategy of the Adunqiaolu site in Xinjiang, China. Quaternary Sciences 2019; 39(1):37–47. 邵孔兰等《植物微体化石分析揭示阿敦乔鲁遗址古人生存策略》. 《第四纪研究》, 第39卷 (第1期), 第37–47页.

[53] Rhodes TE, Gasse F, Lin Ruifen, Fontes JCh, Wei Keqin, Bertrand F, et al A Late Pleistocene-Holocene lacustrine record from Lake Aibi, Zunggar (northern Xinjiang, western China). Paleogeogr, Paleoclimatol, Paleoecol 1996;120:105–121.

[54] Tudryn A, Tucholka P, Gibert E, Gasse F, Wei K. A late Pleistocene and Holocene mineral magnetic record from sediments of Lake Aibi, Dzungarian Basin, NW China. Journal of Paleolimnology 2010;44: 109–121.

[55] Xu H, Zhou K, Lan J, Zhang GL, Zhou XY. Arid Central Asia saw mid Holocene drought. Geology 2019;47(3):255–258. 10.1130/G45686.1

[56] Benecke N., von den Driesch A. Horse exploitation in the Kazakh steppes during the Eneolithic and Bronze Age. In: Levine M., Colin R., Boyle K. editors. Prehistoric steppe adaptation and the horse. Cambridge: McDonald Institute for Archaeological Research; 2003 pp. 69–82.

[57] Fitzhugh W. W. The Mongolian Deer Stone-Khirigsuur Complex: Dating and Organiation of a Late Bronze Age Menagerie. In: Bemmann J., Parzinger H., Pohl E., Tseveendorzh D. Current archaeological research in Mongolia. Bonn: Vor- und Frühgeschichtliche Archäologie Rheinische Friedrich-Wilhelms-Universität; 2009 pp. 183–199.

[58] Wright J. Landscapes of inequality? A critique of monumental hierarchy in the Mongolian Bronze Age. Asian Perspectives 2012;51(2): 139–163.

[59] Gryaznov M. Der Großkurgan von Aržan in Tuva, Südsibirien. München: C.H. Beck; 1984.

[60] Caspari G., Sadykov T., Blochin J., & Hajdas I. Tunnug 1 (Arzhan 0)–an early Scythian kurgan in Tuva Republic, Russia. Archaeological Research in Asia, 2018;15: 82–87.

[61] Wagner M., Wu X., Tarasov P., Aisha A., Ramsey C. B., Schultz M., et al Radiocarbon-dated archaeological record of early first millennium BC mounted pastoralists in the Kunlun Mountains, China. Proceedings of the National Academy of Sciences 2011; 108(38): 15733–15738. 10.1073/pnas.1105273108 PMC3179056 21911387

[62] Sadykov T., Caspari G., & Blochin J. Kurgan Tunnug 1—New Data on the Earliest Horizon of Scythian Material Culture. Journal of Field Archaeology, 2020: 1–15.

